# Tumor-associated macrophages promote ovarian cancer cell migration by secreting transforming growth factor beta induced (TGFBI) and tenascin C

**DOI:** 10.1038/s41419-020-2438-8

**Published:** 2020-04-20

**Authors:** Anna Mary Steitz, Alina Steffes, Florian Finkernagel, Annika Unger, Leah Sommerfeld, Julia M. Jansen, Uwe Wagner, Johannes Graumann, Rolf Müller, Silke Reinartz

**Affiliations:** 1 Institute of Molecular Biology and Tumor Research (IMT), Center for Tumor Biology and Immunology, Philipps University, Marburg, Germany; 2 Clinic for Gynecology, Gynecologic Oncology and Endocrinology, Center for Tumor Biology and Immunology, Philipps University, Marburg, Germany; 3 Clinic for Gynecology, Gynecological Oncology and Gynecological Endocrinology, University Hospital Giessen and Marburg (UKGM), Marburg, Germany; 4 Biomolecular Mass Spectrometry, Max-Planck-Institute for Heart and Lung Research, Bad Nauheim, Germany; 5 The German Centre for Cardiovascular Research (DZHK), Partner Site Rhine-Main, Max Planck Institute for Heart and Lung Research, Bad Nauheim, Germany

**Keywords:** Cancer microenvironment, Ovarian cancer, Experimental models of disease

## Abstract

A central and unique aspect of high-grade serous ovarian carcinoma (HGSC) is the extensive transcoelomic spreading of tumor cell via the peritoneal fluid or malignant ascites. We and others identified tumor-associated macrophages (TAM) in the ascites as promoters of metastasis-associated processes like extracellular matrix (ECM) remodeling, tumor cell migration, adhesion, and invasion. The precise mechanisms and mediators involved in these functions of TAM are, however, largely unknown. We observed that HGSC migration is promoted by soluble mediators from ascites-derived TAM, which can be emulated by conditioned medium from monocyte-derived macrophages (MDM) differentiated in ascites to TAM-like asc-MDM. A similar effect was observed with IL-10-induced alternatively activated m2c-MDM but not with LPS/IFNγ-induced inflammatory m1-MDM. These observations provided the basis for deconvolution of the complex TAM secretome by performing comparative secretome analysis of matched triplets of different MDM phenotypes with different pro-migratory properties (asc-MDM, m2c-MDM, m1-MDM). Mass spectrometric analysis identified an overlapping set of nine proteins secreted by both asc-MDM and m2c-MDM, but not by m1-MDM. Of these, three proteins, i.e., transforming growth factor beta-induced (TGFBI) protein, tenascin C (TNC), and fibronectin (FN1), have been associated with migration-related functions. Intriguingly, increased ascites concentrations of TGFBI, TNC, and fibronectin were associated with short progression-free survival. Furthermore, transcriptome and secretome analyses point to TAM as major producers of these proteins, further supporting an essential role for TAM in promoting HGSC progression. Consistent with this hypothesis, we were able to demonstrate that the migration-inducing potential of asc-MDM and m2c-MDM secretomes is inhibited, at least partially, by neutralizing antibodies against TGFBI and TNC or siRNA-mediated silencing of TGFBI expression. In conclusion, the present study provides the first experimental evidence that TAM-derived TGFBI and TNC in ascites promote HGSC progression.

## Introduction

Ovarian carcinoma is the most lethal gynecological cancer with an overall 12-year survival rate of <20%, and represents the fifth leading cause of cancer-associated deaths in females^1^. A hallmark of high-grade serous carcinoma (HGSC), the most common and aggressive subtype, is its extensive peritoneal metastasis, which occurs at a very early stage of disease and contributes to its fatal clinical outcome^2^. Metastatic spreads occurs predominantly to the omentum and serous membranes lining the peritoneal organs through transcoelomic dissemination of tumor cells via the peritoneal fluid^3–5^. The peritoneal tumor microenvironment (TME), which consists of tumor-infiltrated host tissues and peritoneal fluid (or ascites at advanced stages), is an essential determinant of metastatic disease progression. Ascites contains large numbers of cells including tumor spheroids and immune cells, such as tumor-associated macrophages and T cells (TAMs and TATs, respectively)^6–8^, as well as soluble factors and extracellular vesicles released by tumor and host cells^2,9,10^, collectively referred to as the tumor secretome and recognized as a key player in the communication network of the TME^11–13^. A detailed understanding of the secretome composition, the origin of single compounds, and their role in tumor–host crosstalk remains, however, elusive.

TAMs constitute a prominent cell population in ascites known to promote tumor growth, metastasis, and immunosuppression^14–16^. TAMs are reprogrammed by factors of the TME to adopt a pro-tumorigenic and immunosuppressive phenotype, which is linked to a poor clinical outcome^8,13,17,18^. TAMs contribute to the tumor secretome by releasing a plethora of soluble mediators, such as interleukin (IL)-6, IL-10, C-C chemokine motif ligand 18 (CCL18), CCL22, tumor necrosis factor α, and transforming growth factor beta (TGFβ), that trigger pro-tumorigenic signaling pathways in both tumor and host cells of the TME^19–21^. For example, it is suggested that TAM and tumor cells cooperate in extracellular matrix (ECM) remodeling, which is a prerequisite for tumor cell adhesion and invasion^7,13,14^. Consistent with this model, it has been reported that TAM secrete migration-promoting factors like insulin-like growth factor 1 (IGF1), epidermal growth factor (EGF), and CHI3L1 pointing to a presumably central role of TAM in cancer cell migration, adhesion, and invasion^22–24^.

The TAM-derived mediators that promote cancer migration in the context of the HGSC microenvironment remain largely unknown. This is largely due to the fact that macrophages secrete a plethora of soluble factors, thus complicating the identification of relevant mediators. To address this issue, we designed an experimental setting that compares the secretomes of macrophages with different migration-stimulating properties. This was achieved by comparing matched pairs of monocyte-derived macrophages (MDM) from healthy donors that were differentiated into TAM-like MDM by ascites (asc-MDM), alternatively activated M2 by IL-10 (m2c-MDM)^25^ as well as MDM classically activated by lipopolysaccharide (LPS) and interferon-γ (IFNγ) (m1-MDM). While asc-MDM and m2c-MDM share the potential to stimulate HGSC cell migration similarly to patient-derived TAMs, m1-MDM has no migration-promoting potential.

By combining mass spectrometry (MS)-based proteomics, bioinformatic analyses, and tumor migration assays, we found three candidates with migration-promoting properties released by both asc-MDM and m2c-MDM, but not by m1-MDM. These secreted proteins were transforming growth factor beta induced (TGFBI), tenascin C (TNC), and fibronectin (FN1), which have in common that they are ECM proteins and as such may provide support for tumor cell adhesion and migration. In general, excessive synthesis and deposition of ECM proteins is a hallmark of the tumor stroma, which is especially mediated not only by carcinoma-associated fibroblasts (CAFs)^26^ but also by TAM^13,27^. So far, TNC and TGFBI secretion by TAM has not been linked to tumor cell migration. In the present study, we identified TGFBI, TNC, and FN1 in ascites and found correlations with HGSC progression, supporting a potential clinical relevance of these mediators in the TME. For TGFBI and TNC in particular, we provide evidence for enhanced secretion into the TME as a novel mechanism by which TAM promote HGSC migration.

## Results

### Ascites-derived TAM secrete soluble mediators promoting HGSC migration

**Fig. 1:**
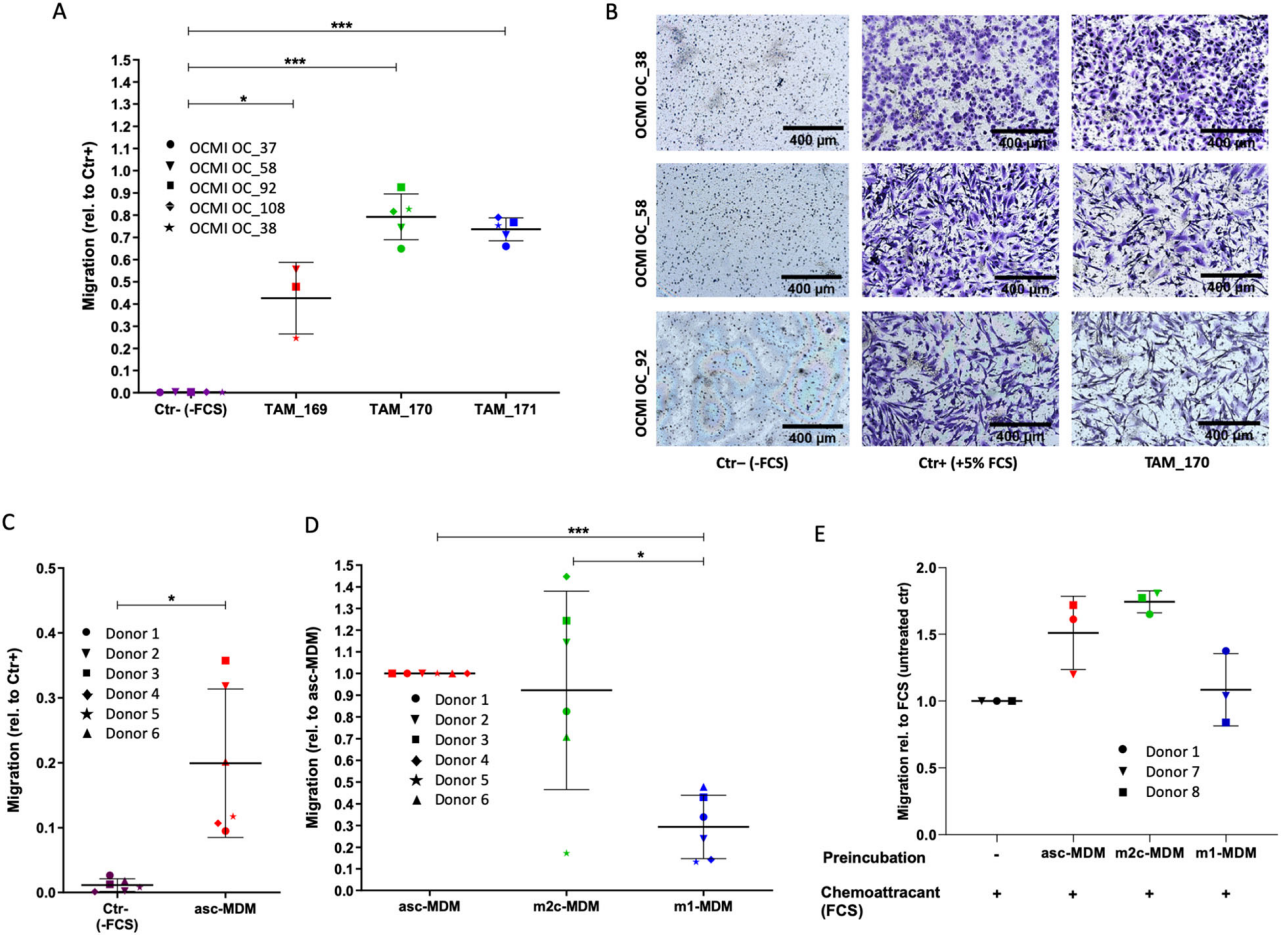
Impact of macrophage secretomes on the migration of ovarian cancer cells. **a** Migration of 5 different cultured patient-specific HGSC tumor cells (OCMI OC_37, 38, 58, 92, 108) was analyzed using conditioned media of ascites-derived TAM from 3 different patients (TAM_169, 170, 171) as chemoattractant in a transwell assay format. Background migration was measured in the absence of any attractant (Ctr−). Data were normalized to 1 for FCS-induced migration (Ctr+) in each OCMI cell line (**a**). **b** Exemplary microscopic pictures showing migrated OCMI cells (OC_ 38, 58, 92) in response to conditioned media of TAM_170 and FCS (Ctr+) as well as background control without chemoattractant (Ctr−). **c**, **d** Conditioned media of m1 (induced by LPS+IFNγ), m2c (induced by IL-10), and asc-MDM (induced by ascites) from 6 donors were applied as attractants for migration of OCMI cell line OC_58. The corresponding data for the phenotypes of MDM differentiation are shown in Supplementary Fig. S1. Migration is expressed relative to FCS-induced migration (**c**) and relative to migration induced by TAM-like MDM (**d**). **e** Transwell migration format using OCMI cells (OC_58) pretreated with conditioned media of m1-MDM, m2c-MDM, and asc-MDM (3 different donors) for 17 h prior to analysis of tumor migration using FCS as chemoattractant for 2 h. As controls, untreated tumor cells were allowed to migrate in the presence (Ctr+) and absence of FCS (Ctr−). For details, see “Materials/subjects and methods.” Migration of pretreated tumor cells was expressed relative to untreated cells in the presence of FCS. Horizontal bars show the mean. Standard deviations are given. Asterisks indicate *p* values determined by two-sided, paired *t* test. *p < 0.05, ***p < 0.001.

As shown in Fig. 1, the secretome of ascites-derived TAM induced strong migration of cultured patient-derived HGSC cells (termed OCMI cells) when applied as chemoattractant in a transwell assay. These findings were validated using conditioned media of TAM from three patients and tumor cells from five patients, indicating that ascites-derived TAM secrete migration-promoting mediators acting on different OCMI tumor cells (Fig. 1a, b).

The highly complex composition of the TAM secretome, which consists of several hundred proteins, complicates the identification of the pro-migratory mediators that are relevant within the HGSC microenvironment. We therefore designed an experimental approach suitable to selectively identify the TAM-derived mediators able to promote HGSC cell migration (see also “Introduction”). This approach is based on our observation that MDM differentiated in vitro with malignant ascites into TAM-like MDM (asc-MDM) emulated the pro-migratory potential of the patients’ TAM secretomes. This was demonstrated in a transwell migration assays, where conditioned medium from asc-MDM significantly induced tumor cell migration when used as chemoattractant (Fig. 1c and Supplementary Fig. S1). A similar impact on migration was observed by conditioned medium from MDM alternatively activated by IL-10 (m2c-MDM), whereas migration was not significantly affected by conditioned medium MDM skewed to a pro-inflammatory phenotype by LPS and IFNγ (m1-MDM) (Fig. 1d and Supplementary Fig. S1). These observations were confirmed in a second migration assay format, where conditioned media was used for pre-incubating tumor cells (prior to the migration assay) rather than as chemoattractant. As shown in Fig. 1e, tumor cells that were pre-incubated with conditioned media of asc-MDM and m2c-MDM, but not of m1-MDM, exhibited increased migratory potential (Fig. 1e).

The MDM differentiation phenotypes were also verified by flow cytometry (see Supplementary Fig. S2). TAM-like asc-MDM were characterized by an increased expression of CD14, CD16, and the m2c markers CD163 and CD206, as well as downregulated expression of the M1 markers CD86 and CCR7 in all tested donors (relative to m1-MDM; Fig. S2).

The differential effects of asc-MDM and m2c-MDM on the one hand and m1-MDM on the other hand paved the way for a detailed comparative analysis of the corresponding secretomes aiming at the identification of candidate proteins with a pro-migratory function secreted by asc-MDM and m2c-MDM (and by TAM in malignant ascites) but not by m1-MDM.

### Comparative secretome analysis of MDM subtypes identifies candidates related to tumor migration

**Fig. 2:**
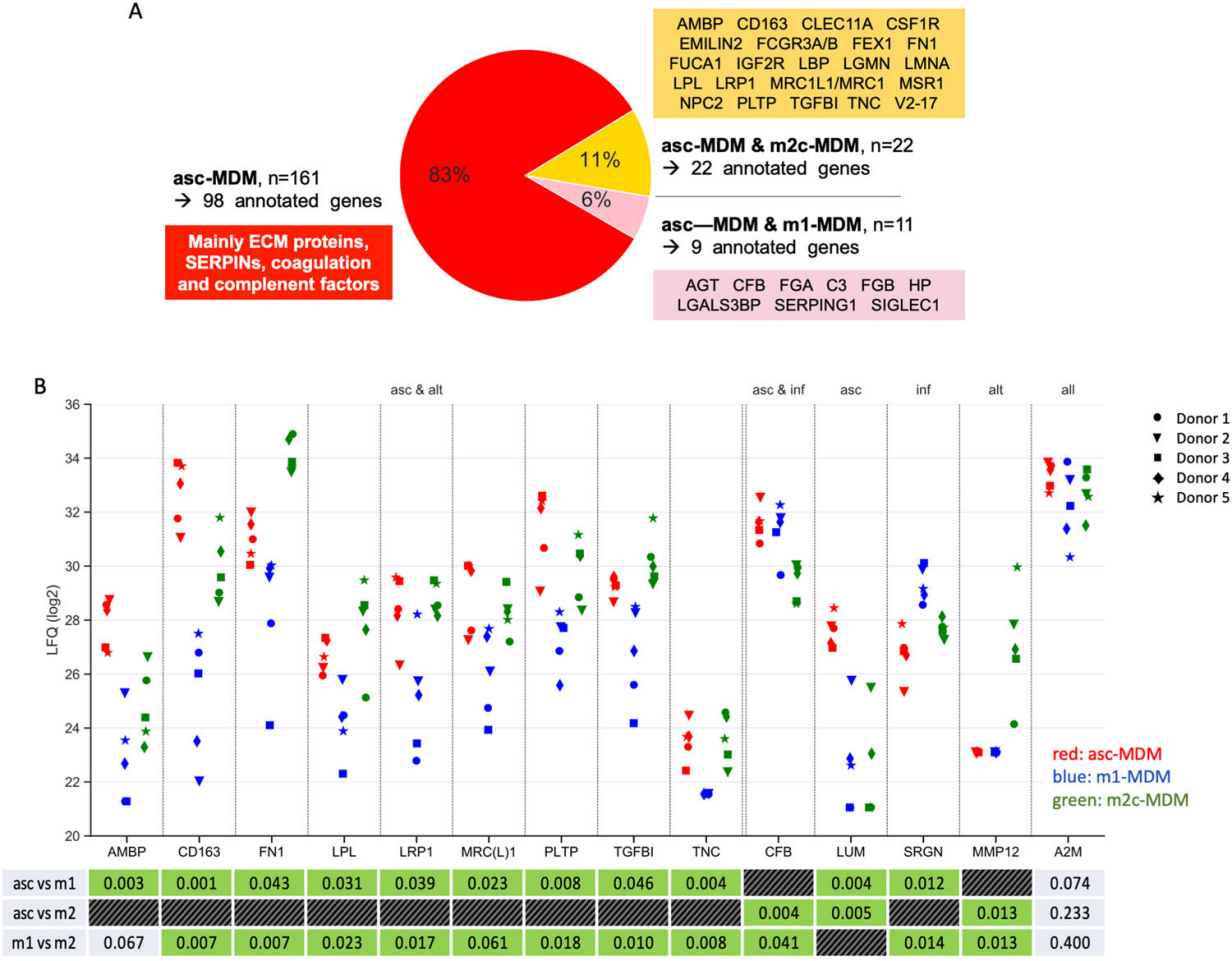
Secretome analysis of MDM subtypes by LC-MS/MS. Serum-free conditioned media of m1-MDM (induced by LPS+IFNγ), m2c-MDM (induced by IL-10), and asc-MDM (induced by ascites) from the same 5 donors tested for stimulation of tumor cell migration in Fig. 1 were analyzed by mass spectrometry-based proteomics. **a** Pie chart showing the distribution of proteins present selectively in the medium from asc-MDM and m2c-MDM versus m1-MDM (orange), asc-MDM and m1-MDM versus m2c-MDM (pink), and asc-MDM versus m1-MDM and m2c-MDM (red). Numbers (*n*) refer to the identified polypeptides (feature_ids in Table S1); arrows point to the number of annotated genes that could be associated with the identified polypeptides. The respective genes (or gene functions) are listed in the colored boxes. **b** Dot plot showing protein levels individually (log_2_ LFQ values measured by LC-MS/MS) for MDM from the same donors as in Fig. 1. Arrows indicate the following selectivities: asc & alt: asc-MDM and m2c-MDM versus m1-MDM (orange in **a**); asc & inf: higher level found with asc-MDM and m1-MDM versus m2c-MDM (pink in **a**); asc: asc-MDM versus m1-MDM and m2c-MDM (red in **a**); inf: m1-MDM versus asc-MDM and m2c-MDM; alt: m2c-MDM versus m1-MDM and m2c-MDM. The table at the bottom shows *p* values (paired *t* test) for the relevant comparisons. Green: *p* < 0.05; gray *p* ≥ 0.05.

Comparative analysis of conditioned media from functionally different MDM subtypes was performed by MS-based proteomics. In total, we identified 700 proteins annotated as “predicted secreted” in the Human Protein Atlas in the supernatant of at least one asc-MDM sample (Table S1). Of these, the 22 proteins were present at higher levels in conditioned media from asc-MDM and m2c-MDM compared to m1-MDM in at least four out of five triplets (Fig. 2a; Tables 1 and S2). Nine of these proteins (AMBP, CD163, FN1, LPL, LRP1, MRC1L1/MRC1, PLTP, TGFBI, TNC) perfectly fit this distribution in all five triplets (Fig. 2b). Among these, three proteins are migration-promoting candidates as suggested by literature data^28–30^, i.e., TGFBI, TNC, and FN1 (Table 1). A similar observation was made for macrophage mannose receptor 1 (MRC1, CD206) and scavenger receptor cysteine-rich type 1 protein (CD163), which are known to be commonly upregulated in TAM and alternatively activated macrophages^8,17,25^, and thus serve as plausibility controls.

**Table 1:** Top 22 secreted proteins overexpressed in the secretomes of asc-MDM and m2c-MDM compared to m1-MDM.

Gene name	Protein name	Match (*n*)^a^
AMBP	Alpha-1 microglobulin	5
CD163	Scavenger receptor CD163	5
FN1	Fibronectin	5
LPL	Lipoprotein lipase	5
LRP1	LDL receptor-related protein 1	5
MRC(L)1	Macrophage mannose receptor 1	5
PLTP	Phospholipid transfer protein	5
TGFBI	Transforming growth factor beta induced	5
TNC	Tenascin	5
IGF2R	Cation-independent mannose-6-phosphate receptor	4
CLEC11A	C-type lectin domain family 11 member A	4
CSF1R	Macrophage colony-stimulating factor 1 receptor	4
EMILIN2	Elastin Microfibril Interfacer 1	4
FCGR3A	Fc fragment of IgG receptor IIIA	4
FUCA1	Alpha-1 fucosidase	4
LBP	Lipopolysaccharide-binding protein	4
LGMN	Legumain	4
LMNA	Lamin A/C	4
MSR1	Macrophage scavenger receptor 1	4
NPC2	Epididymal secretory protein E1	4
STAB1 (FEX1)	Stabilin	4
V2-17	Ig lambda chain V–IV region Hil	4

^a^Match (*n*) = number of donors matching the classification of selectivity (*n* out of 5).

We also identified proteins selective for other MDM subtypes, including 9 proteins with annotated genes for asc-MDM and m1-MDM versus m2c-MDM (Table S3; Fig. 2a), as well as 98 proteins for asc-MDM versus both m1-MDM and m2c-MDM (Table S4; Fig. 2a). This is exemplified in Fig. 2b by lumican (LUM), serglycin (SRGN), and metallopeptidase 12 (MMP12), which are secreted proteins selective for asc-MDM, m1-MDM, or m2c-MDM. In contrast, alpha-2-macroglobulin (A2M) is a protein present at similar levels in conditioned media from all macrophage subtypes (Fig. 2b).

Intriguingly, the proteins secreted selectively by asc-MDM are mainly composed of ECM-associated polypeptides (such as collagens, BCAM, LUM, SERPIN protease inhibitors) as well as complement factors (Table S4; Fig. 2a). This is consistent with previous reports describing these proteins as a hallmark of TAM in HGSC ascites^7,13^, further validating the experimental approach.

### TGFBI, TNC, and FN1 are secreted by ascites TAM in vivo and are associated with a short relapse-free survival (RFS)

**Fig. 3:**
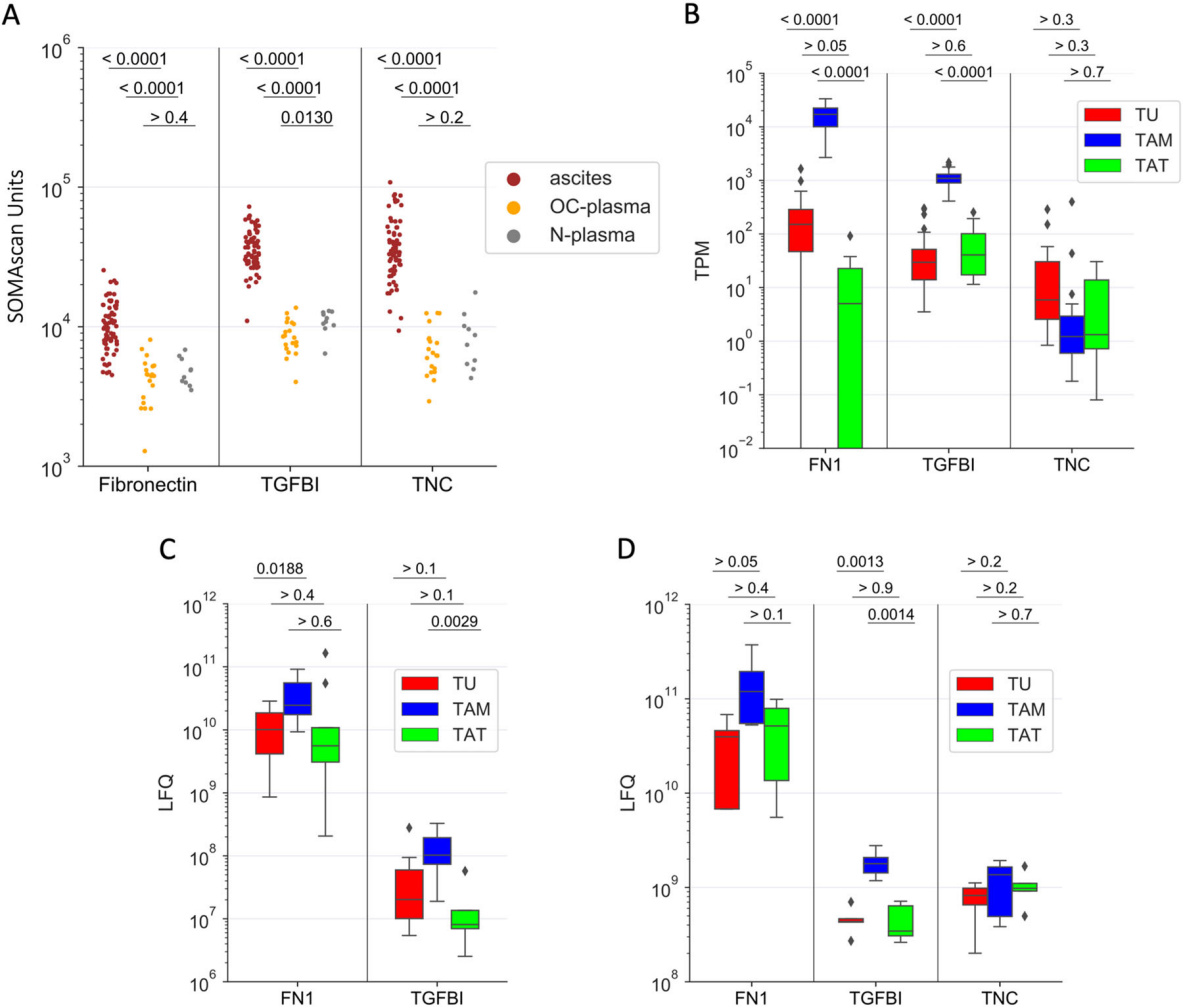
Expression of TGFBI, TNC, and FN1 in malignant ascites and ascites-associated cells. **a** Levels (LC-MS/MS, LFQ intensity) of TGFBI, TNC, and FN1 in cell-free HGSC ascites (*n* = 70, red dots), plasma from HGSC patients (n = 20; OC-plasma, yellow), and patients with benign gynecologic diseases (*n* = 10; N-plasma, gray) as determined by SOMAscan^31^. **b** Expression levels (RNA-Seq, TPM values) for *TGFBI*, *TNC*, and *FN1* in ascites-associated tumor cells (TU *n* = 23, depicted in red), TAM (*n* = 32; blue), and TATs (*n* = 8; green)^7^. **c** Intracellular protein levels (LFQ intensity) of TGFBI, TNC, and FN1 in tumor cells (TU), TAM, and TATs from HGSC patients as obtained from LC-MS/MS-based proteome analysis (*n* = 5 for each cell type)^7^. **d** Levels of TGFBI, TNC, and FN1 (LFQ intensity) in the conditioned media of primary tumor cells (TU), TAM, and TATs after a 5-h cultivation in protein-free medium (*n* = 5 for each cell type). Boxplots show medians (horizontal line in boxes), upper and lower quartiles (box), and range (whiskers) (**b**–**d**). Statistical analyses were performed by unpaired *t* test; *p* values are shown at the top of each panel.

To assess the clinical significance of TGFBI, TNC, and FN1, we analyzed their levels in ascites from 70 HGSC patients and 30 blood plasma samples in our recently published dataset^31^ obtained by the aptamer-based SOMAscan technology^32^. All three proteins were present at significantly higher levels in ascites compared to plasma from patients of the same cohort (*n* = 20) as well as plasma from patients with benign gynecological diseases (*n* = 10) (*p* < 0.0001, Fig. 3a). To elucidate the origin of TGFBI, TNC, and FN1 in HGSC ascites, we made use of our transcriptome, proteome, and secretome datasets for tumor cells, TAMs, and TATs^7,33^. As shown in Fig. 3 for both TGFBI and FN1, RNA expression, intracellular protein levels, as well as secretion were consistently strongest in TAMs compared to tumor cells and TATs (Fig. 3b–d). These findings are consistent with the data reported in the present study showing that asc-MDM secrete TGFBI and FN1. TNC, on the other hand, is also secreted by TAMs at a level comparable to TGFBI (Fig. 3d), but in the corresponding omics datasets *TNC* mRNA was very low in TAM (Fig. 3b) and intracellular TNC protein was not detectable (Fig. 3c). This apparent discrepancy was confirmed with in vitro differentiated asc-TAM by quantitative reverse transcription polymerase chain reaction (qRT-PCR) and western blotting (see below and Fig. 5d–g), which may be explained by an unusual instability of *TNC* mRNA in macrophages combined with rapid protein secretion.

**Fig. 4:**
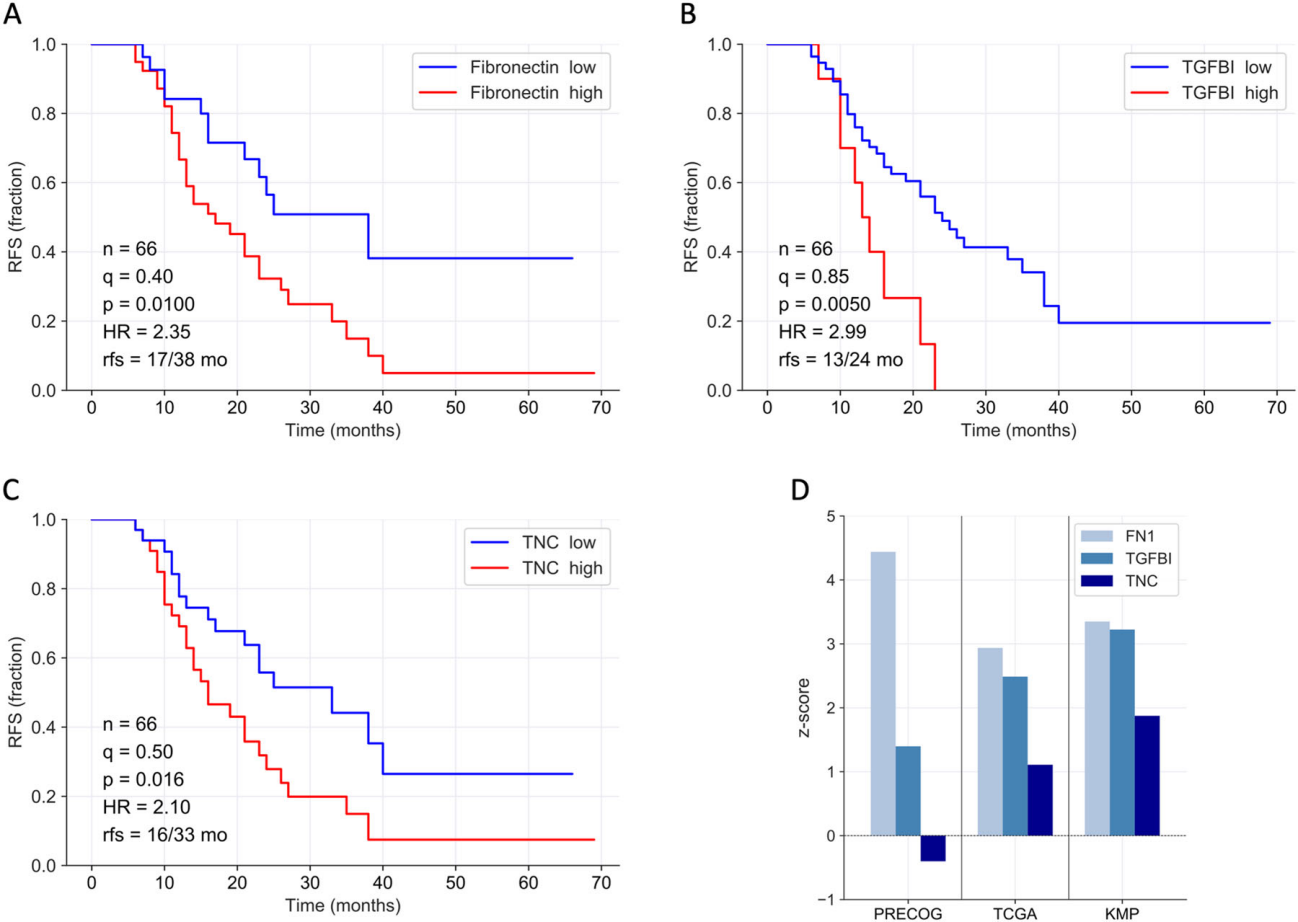
Association of TGFBI, TNC, and FN1 ascites levels with ovarian cancer survival. **a**–**c** Kaplan–Meier plots showing the relationship between relapse-free survival (RFS) and SOMAscan protein signals for fibronectin (**a**), TGFBI (**b**), and TNC (**c**) in cell-free ascites from HGSC patients. *n*: number of evaluable patients; *q* quantile used for splitting datasets (high versus low levels), *p* logrank *p* value, HR hazard ratio, rfs median RFS (months). **d** Mean *z*-scores for survival associations with *TGFBI*, *TNC*, and *FN1* gene expression in solid tissue from ovarian carcinoma) based on public datasets. TCGA^34^ and KMP^35^: relapse-free survival (RFS); PRECOG^36^: overall survival (OS). Positive and negative *z*-scores indicate HR > 1 and HR < 1, respectively. A *z*-score of 1.96 corresponds to a logrank *p* value of 0.05.

Next, we evaluated the potential clinical significance of TGFBI, TNC, and FN1 by associating their ascites levels (SOMAscan data as above) and RFS in a set of 66 HGSC patients. As illustrated in Fig. 4, Kaplan–Meier plots revealed a significant association with high ascites concentrations of TGFBI (Fig. 4a; logrank *p* = 0.010; hazard ratio (HR) = 2.35), TNC (Fig. 4b; *p* = 0.005; HR = 2.99), or FN1 (Fig. 4c; *p* = 0.016; HR = 2.10). These findings are consistent with public datasets (Fig. 4d) showing that the expression of *TGFBI*, *TNC*, and *FN1* mRNA expression in tumor tissue is inversely associated with overall survival (OS) in both database queried, i.e., The Cancer Genome Atlas (TCGA)^34^ and Kaplan–Meier Plotter (KMP)^35^. Both *FN1* and *TGFBI* also showed an association with a short OS in the PRECOG database^36^.

### Validation of TGFBI and TNC secretion by asc-MDM and m2c-MDM

In agreement with our data, TAM isolated from human tumors have been reported to express a matrix-related signature including FN1 affecting tumor cell motility^27^, whereas a role of TGFBI and TNC in the crosstalk between macrophages and tumor cells has not been addressed in previous studies. We therefore focused our work on these two mediators. To validate and extend the proteomics data in Fig. 2b, which showed increased TGFBI and TNC secretion by asc-MDM and m2c-MDM versus m1-MDM, we applied qRT-PCR, western blot, and enzyme-linked immunosorbent assay (ELISA).

**Fig. 5:**
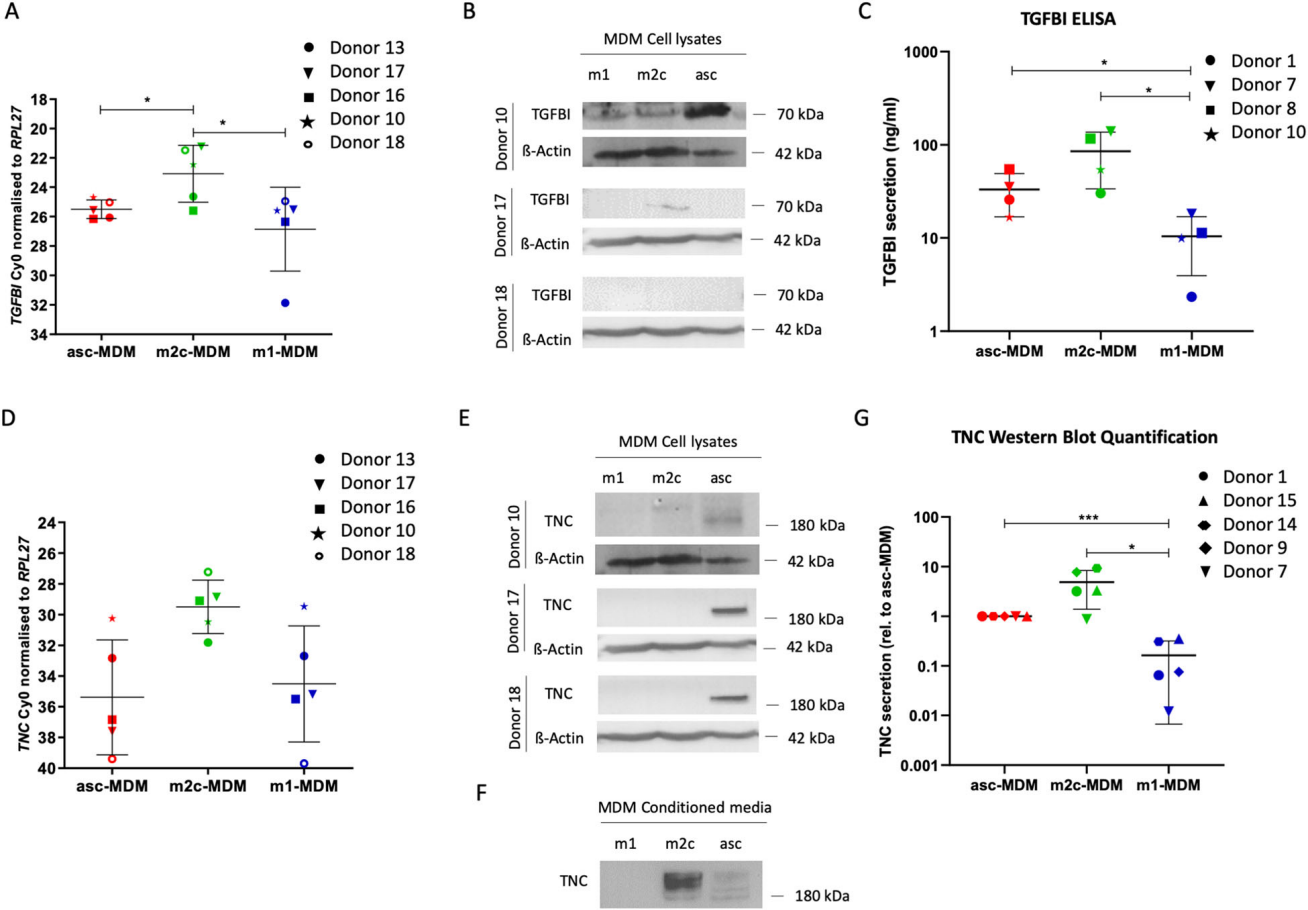
Upregulation of TGFBI and TNC in migration-promoting MDM subtypes. **a** Expression of *TGFBI* mRNA in asc-MDM, m1-MDM, and m2c-MDM analyzed by RT-qPCR in five different donors. **b** Detection of TGFBI protein in cell lysates by western blotting. β-Actin was used as loading control. Blots of three donors are shown. **c** TGFBI secretion of polarized MDM measured by ELISA of conditioned media (*n* = 4). TGFBI protein levels are indicated as ng/ml. **d** Expression of *TNC* mRNA was analyzed in asc-MDM, m1-MDM, and m2c-MDM by RT-qPCR in five different donors. **e** Detection of TNC protein in cell lysates by western blotting. β-Actin was used as loading control (same blot as in **b**, since both TGFBI and TNC were analyzed in the same experiment) (*n* = 3). **f** Western blot of TNC protein in the conditioned media. The analysis was carried out with tenfold concentrated conditioned media from equal numbers of different MDM subtypes. **g** Quantification of TNC secretion by different MDM subtypes was performed using the Image LabTM 5.0 software in five different donors. TNC protein levels were normalized to 1 for asc-TAM. *p* Values were determined by paired *t* test (*p < 0.05, ***p < 0.001).

As illustrated in Fig. 5, *TGFBI* mRNA (Fig. 5a) was significantly higher in m2c-MDM compared to both asc-MDM and m1-MDM but was similar among the latter two MDM subtypes. At the intracellular protein level, TGFBI was weak or even undetectable in m1-MDM and m2c-MDM but higher in asc-MDM in single donors (Fig. 5b). TGFBI secretion was strongest in m2c-MDM but was also elevated in asc-MDM versus m1-MDM (Fig. 5c), which is consistent with the MS data (Fig. 2b). It thus appears that the differences in TGFBI secretion observed among the three MDM subtypes do not solely result from differential regulation of mRNA expression but also from subtype-specific effects on secretion itself.

TNC mRNA expression (Fig. 5d) were similar in asc-MDM and m1-MDM but elevated in m2c-MDM (though *p* > 0.05 due to the very low expression), whereas intracellular TNC was only detectable in asc-MDM (Fig. 5e). As for TGFBI, TNC secretion was also higher in asc-MDM and m2c-MDM versus m1-MDM (Fig. 5f, g), pointing to a similar subtype-dependent regulation of the secretory pathway.

In summary, these analyses fully confirm the MS data and suggest that differential regulation of both gene expression and secretion are responsible for the differences in TGFBI and TNC secretion by MDM subtypes.

### Pro-migratory effects are impaired by neutralizing TGFBI and TNC in the MDM secretome

**Fig. 6:**
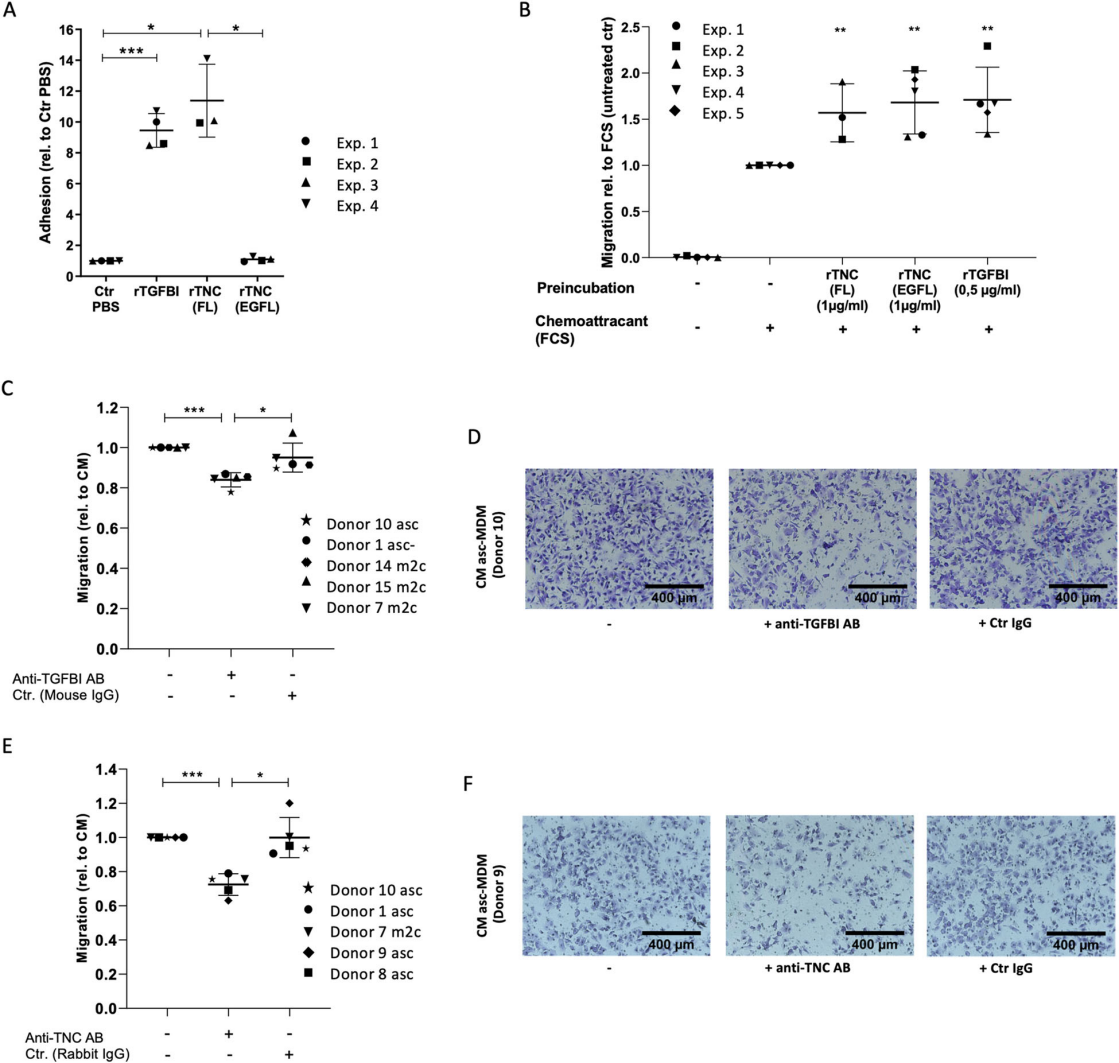
Inhibition of migration-promoting activity of TGFBI and TNC in asc-MDM and m2c-MDM secretomes by neutralizing antibodies. **a** Adhesion of OCMI cells (OC_58) to plastic-coated rTGFBI and rTNC (full-length and EGFL repeat) (or PBS as uncoated control) was analyzed after 1 h. Bound cells were stained with crystal violet, and color development was measured at 560 nm. Adhesion was calculated relative to the uncoated control for each of *n* = 4 experiments. **b** Transwell migration assay format using OCMI cells (OC_58) pretreated with rTGFBI and rTNC (full-length and EGFL repeat) for 17 h. Influence of recombinant proteins on tumor migration was subsequently measured using FCS as chemoattractant for 2 h. As controls, untreated tumor cells were allowed to migrate in the presence (Ctr+) and absence of FCS (Ctr−). Five experiments were performed. Migration of pretreated tumor cells was expressed relative to untreated cells in the presence of FCS. **c**–**f** Neutralization of TGFBI (**c**, **d**) and TNC (**e**, **f**) in conditioned media (CM) of m2c-MDM and asc-MDM (*n* = 5) was performed as described for the recombinant proteins in Supplementary Fig. S1. As a control, the cells were either left untreated or treated with CM without adding the antibodies. Migration was analyzed in the transwell format described above using FCS as chemoattractant. Migration is expressed relative to the migration induced by the CM alone. Horizontal bars show the mean. *p* Values were determined by two-sided, paired *t* test (*p < 0.05, **p < 0.01, ***p < 0.001). Representative microscopic pictures of migrated cells induced by MDM secretomes in the presence and absence of neutralizing anti-TGFBI antibody (**d**) and anti-TNC antibody (**f**) are shown.

We next addressed the impact of TGFBI and TNC secreted by macrophages on tumor migration. TGFBI and TNC both bind to integrins and, in case of TNC, additionally to EGF receptors present on the surface of tumor cells, thereby activating migration-inducing pathways^28,29,37–39^. We could demonstrate that patient-derived tumor cells selectively bind to rTGFBI or rTNC (full-length protein) but not to rTNC-EGFL, which is a smaller fragment harboring EGFL repeats but lacking integrin-binding domains (Fig. 6a). As illustrated in Fig. 6b, rTGFBI as well as both rTNC forms enhanced migration of OCMI cells, which was accomplished by pre-incubating OCMI cells with the recombinant proteins prior to setting up the transwell assay with fetal calf serum (FCS) as chemoattractant. Our findings thus indicate that, in the case of TNC, integrin interaction is required for adhesion but dispensable for migration.

To assess the relevance of TGFBI and TNC in the context of the TAM secretome, we analyzed the effect of neutralizing antibodies directed against these proteins. As a proof of principle, we found that tumor migration induced by rTGFBI and rTNC was blocked by neutralizing anti-TGFBI (Supplementary Fig. S3A, B) and anti-TNC antibodies (Supplementary Fig. S3C, D). More importantly, similar results were obtained when conditioned media from asc-MDM or m2c-DM were pre-incubated with the neutralizing antibodies against TGFBI (Fig. 6c, d) and TNC (Fig. 6e, f). In both cases, a significant reduction of cellular migration compared to untreated or IgG control-treated conditioned media was found for five different donors. In conclusion, these data indicate that TNC and TGFBI as constituents of the TAM secretome promote tumor cell migration.

### Small interfering RNA (siRNA)-mediated TGFBI silencing in m2c-MDM/asc-MDM blocks tumor migration

**Fig. 7:**
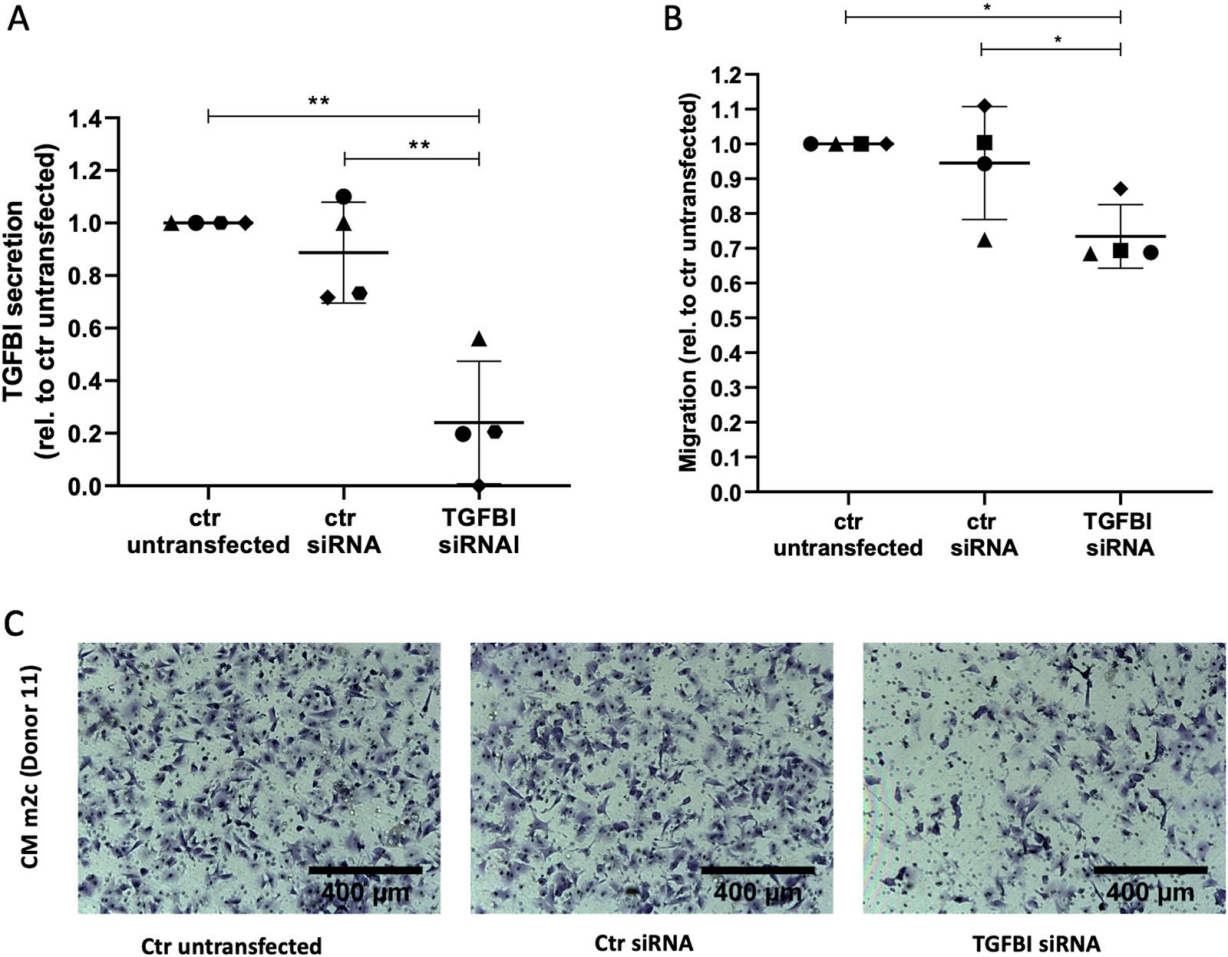
Impact of TGFBI silencing on the migration-promoting potential of asc-MDM and m2c-MDM secretomes. **a** TGFBI secretion by m2c-MDM and asc-MDM after siRNA-mediated TGFBI silencing. TGFBI concentration in the conditioned media of MDM transfected with control siRNA and TGFBI siRNA (pool of three siRNAs) was determined by ELISA and normalized to the untransfected control. Depicted are the data of five different macrophage preparations. Additional data of TGFBI gene expression and intracellular protein levels in TGFBI siRNA-transfected macrophages are shown in Fig. S3. **b** Influence of TGFBI knockdown on the migration-promoting potential of asc-MDM and m2c-MDM. OCMI tumor cells (OC_58) were pretreated with conditioned media of the untransfected and siRNA-transfected cells before applied to a transwell migration assay with FCS as attractant, as described in the legend of Fig. 6. Migration was expressed relative to the untransfected control for each of the four different macrophage preparations. Horizontal bars show the mean and two-sided, paired *t* test was calculated (*p < 0.05, **p < 0.01). **c** Representative microscopic pictures of tumor cell migration induced by conditioned media from m2c-MDM (donor 11) untransfected or transfected with control siRNA or TGFBI siRNA.

To validate our findings by an independent experimental approach, we made use of siRNA-mediated interference. We focused on TGFBI because of the low expression of *TNC* mRNA and TNC protein (Fig. 5d, e), which makes it difficult to reliably monitor silencing efficacy. TGFBI silencing was performed in asc-MDM and m2c-MDM and achieved reduction of *TGFBI* RNA and intracellular TGFBI protein expression by *TGFBI* siRNA transfection relative to control siRNA (Supplementary Fig. S4A, B). Importantly, TGFBI secretion by asc-MDM and m2c-MDM was also inhibited by *TGFBI* siRNA compared to untransfected (*p* < 0.05) and control siRNA-transfected MDM (*p* < 0.01) (Fig. 7a). To investigate the functional impact of TGFBI knockdown on the migration-inducing capacity of asc-MDM and m2c-MDM, transwell assays were performed analogous to the neutralizing experiments above. As shown in Fig. 7b, c, the conditioned media from untransfected or control siRNA-transfected asc-MDM and m2c-MDM induced OCMI cell migration to a very similar extent, whereas transfection with *TGFBI* siRNA resulted in a reduced effect. Taken together, these results establish an essential role for TGFBI as a migration-promoting factor in the TAM secretome.

## Discussion

In the present study we identified TGFBI, TNC, and FN1 as potential mediators of TAM-induced ovarian cancer migration, underscoring the known role of ECM proteins in tumor progression. A clinical importance of all three proteins is supported by our finding that increased ascites level is associated with a short RFS (Fig. 4a–c). Consistent with this observation, we found a similar association of FN1, TGFBI, and TNC gene expression in solid tumor tissue with a poor clinical outcome (Fig. 4d). These findings are in line with studies on colorectal cancer and esophageal squamous cell carcinoma where a poor prognosis correlates with TGFBI expression in tumor stroma^40,41^. For TNC, similar clinical associations have been reported for different tumor entities^42–45^. Furthermore, FN1, TNC, and TGFBI have been reported to promote tumor migration, invasion, and adhesion, which are functions facilitating metastatic spread^28–30^. In this context, FN1 has been proposed as a promoter of ovarian cancer released by CAFs^45^ and TAMs^27^, a hypothesis our results confirm. On the other hand, a mechanistic link of TAM-secreted TNC and TGFBI with tumor migration, as identified in our study, has not been described as of yet.

TGFBI often functions as a linker protein to interconnect ECM molecules and induces cell interactions through integrins^37,46–49^. Different physiological functions including migration and adhesion have been attributed to TGFBI^37,50–52^. Previous work has shown that TGFBI is expressed by stromal fibroblasts and cancer cells^53^. TGFBI upregulation in M2 macrophages has also been linked to acute inflammation processes and ECM remodeling^51^, but TAMs have not been identified as producers of TGFBI to date.

In ovarian cancer and esophageal squamous cell carcinoma, a dual function of TGFBI depending on its cellular origin has been discussed^38,41,54^. Here TGFBI has been proposed to act as tumor suppressor as it is downregulated in tumor cells and as tumor promoter when expressed by peritoneal stroma cells. In accordance with these reports, we demonstrated that TAM secrete higher amounts of TGFBI compared to other cell populations in the ascites, e.g., tumor cells or TATs (Fig. 3), and that TGFBI in the TAM secretome enhances tumor migration proven by neutralizing antibodies (Fig. 6) and RNA silencing (Fig. 7).

Both anti- and pro-adhesive features have been attributed to TGFBI affecting cell motility and invasion. In the case of melanoma, TGFBI exhibits anti-adhesive properties concomitant with anti-migratory activity^55,56^, whereas TGFBI mediates adhesion and migration in renal cell carcinoma^57^. Judging from our data, TGFBI seems to have a pro-adhesive effect in HGSC, since primary OCMI cells adhere strongly to rTGFBI, which is accompanied by enhanced tumor motility (Fig. 6).

Similar to TGFBI, TNC also functions as a modulator of cell adhesion and migration, but a broad range of functions linked to different TNC isoforms (180–330 kDa) have been reported beyond these^58,59^. TNC is downregulated in healthy tissue but transiently re-expressed under pathological conditions like inflammation, wound healing, and cancer^58,60^. Moreover, TNC was found at the invasive front of different tumors with CAFs being the main producers^61–63^. For macrophages, TNC secretion has so far only been shown in atherosclerotic plaques^64^. In the present study, we identify TAM as a cellular origin of TNC in the ovarian TME supporting tumor migration. As shown by western blot, migration-promoting macrophages predominantly secrete large TNC variants of about 200–250 kDa (Fig. 5f), which have been proposed to promote a tumor-supporting TME^59^. Interestingly, a number of studies point to an association between cancer progression and the occurrence of large TNC isoforms harboring alternatively spliced FNIII repeats^59,65^. Alternatively spliced FNIII repeats as well as the RGD-containing FNIII 3 repeat present in TNC isoforms mediate cell adhesion via interaction with different integrins expressed on the surface of tumor cells^58,66–68^. Our data showing that only a full-length rTNC promoted cellular attachment, whereas a smaller fragment lacking integrin-binding sites exhibited anti-adhesive properties (Fig. 6a) may be considered to generally support that assumption. By contrast, both rTNC equally induced tumor migration indicating that the EGFL repeats present in both TNC forms might be involved in tumor migration through activation of EGF receptor signaling.

TGFBI and TNC are both induced by TGFβ signaling and share common binding partners like fibronectin, collagen, and proteoglycans^37,69^. Moreover, both proteins bind to integrins expressed on tumor cells and mediate their function via the integrin signaling pathway^67^. These similarities may contribute to a functional cooperation of TGFBI and TNC in mediating tumor migration, when secreted by TAMs and other cells in the TME.

Other factors, including EGF, IGF1, and CHI3L1, have been proposed to be involved in the migration-promoting function of macrophages^22–24^, but these mediators are not among the proteins upregulated in the pro-migratory MDM secretomes identified by our approach. Several reasons are likely to contribute to these differences.

(i) Our strategy was to identify proteins that are selectively secreted by migration-promoting asc-MDM and m2c-MDM relative to m1-MDM, which do not impact tumor migration. CHI3L1 is actually present in the secretomes from all three MDM subtypes, albeit with elevated levels for m1-MDM (Table S2), which, however, does not preclude a contribution of CHI3L1 to tumor cell migration as seen in other studies^24^.
(ii) The published studies differ from ours in that they use either the THP1 macrophage cell line or MDM differentiated to M2a cells by IL-4, which are likely to secrete different factors.
(iii) We consider only proteins to be relevant that are produced by TAM in vivo. *EGF* and *IGF1* are both not expressed by TAM from HGSC ascites (median transcripts per kilobase million (TPM) < 0.1) or at very low levels (median TPM = 1), respectively (https://www.ovara.net/resources)^7^. Both proteins were neither detectable in the intracellular proteome of TAM nor in their secretome^7^ and were not found in the conditioned medium from asc-TAM (and this study, Table S2). It therefore appears unlikely that EGF and IGF1 play a role in the HGSC TME as proteins secreted by TAMs. However, both proteins are present in the ascites proteome of HGSC patients^31^, pointing to other cell types as producers of EGF and IGF1. Therefore, a role of these proteins in tumor migration and HGSC metastasis cannot be ruled out.

In conclusion, TGFBI, TNC, and FN1, predicted by our experimental model as migration-promoting proteins secreted by TAMs were validated to be (i) present in the HGSC ascites, (ii) secreted by TAMs derived from ascites, (iii) associated with a poor clinical outcome, and (iv) promote tumor migration as part of the TAM secretome. These findings provide further evidence for the essential role of TAMs and the ECM in HGSC metastasis.

## Materials/subjects and methods

### Ascites and cells isolated from ovarian cancer patients

Ascites was collected from untreated patients with HGSC prior to surgery at Marburg University Hospital. The collection and analysis of human material were approved by the ethics committee at Philipps University (reference number 205/10). Donors provided written consent in accordance with the Declaration of Helsinki. TAMs, TATs, and tumor cells were isolated from ascites as previously described^7,33^. Briefly, mononuclear cells were separated by Lymphocyte Separation Medium 1077 (PromoCell, Heidelberg, Germany) density gradient centrifugation followed by MACS separation of CD14+ TAMs and CD3+ TATs and purification of tumor spheroids by size exclusion. Cell-free ascites was cryo-preserved at −80 °C. Permanent primary tumor cell cultures (termed OCMI tumor cells) were established from ascites tumor spheroids according to Ince et al.^70^ with modifications, as previously reported^71^. This culture system allows for the propagation of ovarian cancer cells over long periods of time in the absence of culture-induced crisis or genetic alterations as compared to the original tumor. Cultured HGSC patient-derived OCMI cell lines (OCMI OC_37, OC_38, OC_58, OC_92, and OC_108) were tested for mycoplasma contamination before use for functional analysis.

### Isolation and culture of MDMs from healthy donors

Buffy coats from healthy adult volunteers were kindly provided by the Center for Transfusion Medicine and Hemotherapy at the University Hospital Gießen and Marburg, and mononuclear cells were isolated by Ficoll density gradient centrifugation. CD14+ monocytes were purified by adherence selection and used for subsequent differentiation at a concentration of approximately 2.5 × 10^6^ cells per 6-well plate. For differentiation into TAMs like asc-MDM, monocytes were cultured in cell-free ascites pool derived from 5 patients for 7 days. m1-MDM and m2c-MDM were generated by culturing monocytes in RPMI1640 (Life Technologies, Darmstadt, Germany) supplemented with 5% human AB serum (Sigma), 1% sodium pyruvate (Sigma Aldrich, Taufkirchen, Germany), and 100 ng/ml granulocyte macrophage colony-stimulating factor (CSF) (Peprotech, Hamburg, Germany) for m1-MDM or 20 ng/ml macrophage CSF (M-CSF; Biolegend, San Diego, CA, USA) for m2c-MDM^25^. After 5 days, 100 ng/ml LPS (Sigma Aldrich, Taufkirchen, Germany) and 20 ng/ml IFNγ (Biozol, Echingen, Germany) was added for m1-MDM and 20 ng/ml IL-10 (Biozol, Echingen, Germany) for m2c-MDM activation for 2 days.

### Flow cytometry

The differentiation phenotype of MDM was characterized by flow cytometry (FACSCanto II BD Biosciences) as described previously^8^ using the following antibodies for surface staining: anti-human CD14-FITC (5170518160, Miltenyi Biotec, Bergisch Gladbach, Germany), CD86-FITC (5170620163, Miltenyi Biotec), CD16-PE-Cy7 (4273442, eBioscience, Frankfurt, Germany), CD163-PE (4303842, eBioscience), HLA-DR-APC (4330406, eBioscience), CCR7-PE (5247917, BD Biosciences, Heidelberg, Germany), and CD206-APC (B202691, Biolegend). Corresponding isotype-matched controls were purchased from Miltenyi Biotec (5161221581; 5161017246) and BD Biosciences (6286946; 25471442). The gating for macrophages was performed based on the surface expression of CD14 marker. Results were calculated as the percentage of positive cells and mean fluorescence intensities.

### Generation of conditioned media

For the proteomic analysis in Fig. 3, conditioned media from ascites-derived tumor spheroids, TAMs, and TATs were generated as described by Worzfeld et al.^7^. Conditioned media of TAMs were also used for tumor migration assays (Fig. 1). Therefore, freshly prepared TAMs were cultured in autologous cell-free ascites (or ascites pool of five patients) at a density of 2.5 × 10^6^ cells per 6-well plate for 16 h at 37 °C and 5% CO_2_. Thereafter, the ascites was aspirated, and the cells were washed three times in phosphate-buffered saline (PBS) and twice in serum-free media M199 (Gibco, Thermo Fisher Scientific, Schwerte, Germany) mixed 1:2 with Dulbecco’s Modified Eagle’s Medium/Ham’s F-12 (1:1 Biochrom, Berlin, Germany). TAMs were cultured in medium (750 μl per 6-well) without ascites or serum for another 5 h at 37 °C and 5% CO_2_ before collecting the conditioned media for secretome analysis and functional testing. This time point was chosen as prolonged incubation of TAMs resulted in increased cell death as shown by lactate dehydrogenase release. Conditioned media from differentiated MDM were obtained analogously, except that MDM were cultured for 18 h in serum-free medium. For immunoblotting, conditioned media were concentrated tenfold using a vacuum concentrator.

### Proteomic and transcriptomic analyses

Cell culture supernatants from MDM cultures was obtained as described above. Up to 40 µg of proteins were loaded on a gradient gel (NuPAGE 4–12% Bis-Tris gel, Invitrogen, Carlsbad, CA, USA), separated by sodium dodecyl sulfate-polyacrylamide gel electrophoresis prior to in-gel digestion^72^ and analyzed by liquid chromatography tandem MS/MS as previously reported^7^. The proteomics data have been deposited to the ProteomeXchange Consortium via the PRIDE partner repository^73^ at www.ebi.ac.uk/pride/archive (dataset identifier PXD016555). Data were processed as described^7^ using the human uniProt database (canonical and isoforms, downloaded on 02/09/2018, 183579 entries). Relevant parameters for instrumentation extracted using MARMoSET^74^ and are, along with MaxQuant^75–77^ (v. 1.6.1.0) parameters, included in Supplementary Materials. Transcriptomic and proteomic data for TAMs, TATs and tumor cells from ascites were derived from our published datasets^7,33^.

### Identification of secreted proteins selective for MDM subtypes

MS data were filtered to include only proteins detected in at least 1 of the 5 asc-MDM samples with a minimum log_2_ LFQ of 22 (corresponding to the median of the entire dataset with missing values replaced by imputation). Differences between asc-MDM, m1-MDM, and m2c-MDM were determined for each protein and triplet. Proteins were considered subtype-selective if they were present in the medium from one MDM subtype at a higher level than in the culture supernatant from another subtype in at least four out of the five triplets.

### Tumor cell migration

Transwell migration assays were performed using two different formats. In a first approach, the migration of primary OCMI tumor cells was determined in the presence of conditioned media of macrophages or recombinant human rTGFBI (R&D Systems, Wiesbaden, Germany) and rTNC (fragment containing the EGFL repeats: R&D Systems; full-length protein: Merck, Darmstadt, Germany) as chemoattractant. 50,000 tumor cells were seeded in 300 µl serum-free OCMI medium per transwell insert (8.0 µm pore size; BD Biosciences). Conditioned media of macrophages (1:3 diluted in serum-free OCMI) and rTGFBI (0.5-5 μg/ml) and rTNC (1-10 μg/ml) (or 5% FBS as positive control) in serum-free OCMI medium were added as chemoattractants to the lower chamber. The cells were allowed to migrate through the filter for 17 h at 37 °C in a 5% CO_2_ incubator. Filters were stained with crystal violet solution (0.2% in 20% methanol, 1:5 dilution) for 10 min and evaluated under a Leica DMI3000B microscope (Leica, Wetzlar, Germany). Migrated cells were counted in seven visual fields per filter using the ImageJ software. In a second setting, OCMI tumor cells were pre-incubated with conditioned media of macrophages (1:3 diluted in OCMI medium) and recombinant proteins for 17 h at 37 °C and 5% CO_2_ prior to performing transwell migration assays with 5% FCS as chemoattractant. Where indicated, neutralizing antibodies (10 µg/ml) directed against TGFBI (3054632, Proteintech, Manchester, UK) and TNC (10000035, Merck)—or equivalent amounts of species-matched rabbit (I5006-10MG, Sigma Aldrich) and mouse IgG (131515, Jackson Immuno Research, Cambridgeshire, UK) as controls—were added to conditioned macrophage medium or recombinant proteins for 1 h before applying to the tumor cells. In each case, the pretreated tumor cells were allowed to migrate for 2 h and analyzed as described above.

### Tumor cell adhesion to TNC and TGFBI

Ninety-six-well plates were coated in triplicates with 10 μg/ml rTGFBI and rTNC in PBS (or PBS alone as negative control) overnight at 4 °C. Wells were blocked with 1% bovine serum albumin in PBS for 1 h at 37 °C and washed three times with PBS. Fifty thousand tumor cells in OCMI media were added per well and allowed to adhere for 2 h at 37 °C. The wells were washed with PBS twice to remove any unbound cells. Adherent cells were fixed with glutaraldehyde and stained with 0.1% crystal violet as described^78^. The photometric measurement was performed at 560 nm, and cell adhesion was expressed relative to the negative control.

### TGFBI quantification by ELISA

TGFBI concentrations in conditioned media of macrophages were quantified by commercial ELISA (human βIG-H3 ELISA duo set, R&D Systems, Wiesbaden, Germany) according to the instructions of the manufacturer.

### Immunoblotting and protein quantification

Immunoblots were performed according to standard western blotting protocols using the following antibodies: α-TGFBI (5601, Cell Signaling, Danvers, MA, USA), α-TNC (10000035, Merck), α-β-Actin (A5441, Sigma Aldrich), α-rabbit IgG horseradish peroxidase (HRP)-linked AB (27, Cell Signaling), and α-mouse IgG HRP-linked AB (32, Cell Signaling). Imaging and quantification was done using the ChemiDoc MP system and Image Lab software version 5 (Bio-Rad, Hercules, CA, USA).

### RNA isolation and RT-qPCR

cDNA isolation and qPCR analyses were performed as described previously^79^. L27 was used for normalization. RT-qPCR was carried out using the following primers: RPL27, AAAGCTGTCATCGTGAAGAAC and GCTGTCACTTTGCGGGGGTAG; TGFBI, AAAGACATCCTAGCCACCAACG and AGCTGGCCTCTAAGTATCTGTACC; and TNC, GCCTCCACAGCCAAAGAACC and TCTGGTGCTGAACGAACTGC. Raw data were evaluated with the Cy0 method^80^.

### siRNA transfection of macrophages for transient TGFBI knockdown

siRNA transfection was performed in m2c-MDM and TAM-like differentiated asc-MDM as well as ascites-derived TAMs according to the manufacturer’s protocol using the TransIT-X2 reagent from Mirus (Madison, WI, USA). The following equimolar mixtures of three siRNA oligonucleotides each from Sigma Aldrich (Taufkirchen, Germany) were used for transfection: siRNA TGFBI #1 (HA12627314; HA12627315), siRNA TGFBI #2 (HA12627318; HA12627319), and siRNA TGFBI #3 (HA12627316; HA12627317). MISSION siRNA Universal Negative Control # 2 from Sigma Aldrich was used as a control siRNA (si-ctrl). For m2c-MDM, transfection was performed in RPMI/5% AB-media containing M-CSF and IL-10. Since ascites interferes with siRNA transfection, ascites-containing culture medium in asc-MDM or TAM was replaced by RPMI/5% AB-media during transfection. In this case, after 6 h transfection medium was changed and ascites was added again to maintain the TAM-like phenotype. Cells were harvested 48 h after transfection for analysis of RNA/protein expression and generation of conditioned media for functional assays.

### Statistical analysis

Comparative data were statistically analyzed by unpaired (Fig. 3) or paired Student’s *t* test (Figs. 1, 2, 5–7) (two-sided, equal variance). Significance levels are indicated as four (****), three (***), double (**) and single (*) asterisks for *p* < 0.0001, *p* < 0.001, *p* < 0.01, and *p* < 0.05, respectively. Box pots in Fig. 3 depicting medians (line), upper and lower quartiles (box), range (whiskers), and outliers/fliers (diamonds) were constructed using the Seaborn boxplot function with Python. Associations with RFS (logrank test), HR, and median survival times were analyzed using the Python Lifelines KaplanMeierFitter and CoxPHFitter functions. All logrank test results are presented as nominal *p* values. The data in Fig. 4d were obtained from the PRECOG and KMP meta-analysis databases^35,36^ and TCGA^34^.

## Supplementary information

Supplementary Tables S1-S5

Supplementary Tables Legends S1-S5

Figure S1

Figure S2

Figure S3

Figure S4

Supplementary Figure Legends S1-S4
