# Supplementary material for: Tumor-associated macrophages promote ovarian cancer cell migration by secreting transforming growth factor beta induced (TGFBI) and tenascin C
Source: Cell Death Dis. 2020 Apr 20;11(4):249. doi: 10.1038/s41419-020-2438-8 (PMC7171168; doi:10.1038/s41419-020-2438-8)
Supplement: Supplementary file 2 — Supplementary Tables Legends S1-S5 [file 41419_2020_2438_MOESM2_ESM.docx]

**Supplementary Table Legends**

**Table S1:**

Proteins found in conditioned media from asc-MDM and annotated as "predicted secreted" in the Human Protein Atlas. The Table shows the log2 LFQ values for these proteins in conditioned media from asc-MDM, m1-MDM and m2c-MDM (5 replicates: R1 - R5)

**Table S2:**

Proteins higher in conditioned media from asc-MDM and m2c-MDM relative to m1-MDM

**Table S3:**

Proteins higher in conditioned media from asc-MDM and m1-MDM relative to m2c-MDM

**Table S4:**

Proteins higher in conditioned media from asc-MDM relative to m1-MDM and m2c-MDM

**Table S5:**

Data for Figure 2B.
