# Supplementary figures and images for: Tumor-associated macrophages promote ovarian cancer cell migration by secreting transforming growth factor beta induced (TGFBI) and tenascin C
Source: Cell Death Dis. 2020 Apr 20;11(4):249. doi: 10.1038/s41419-020-2438-8 (PMC7171168; doi:10.1038/s41419-020-2438-8)

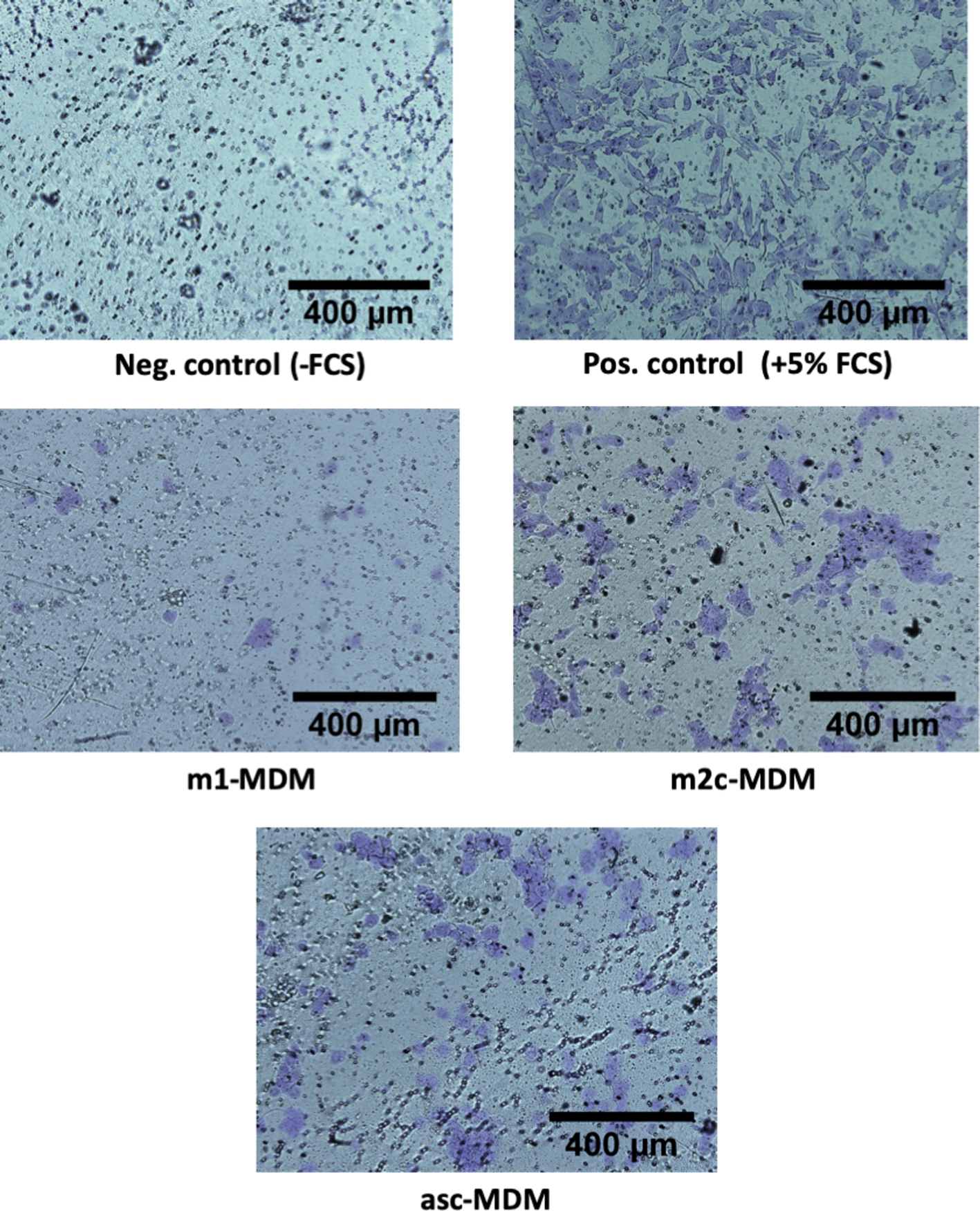

Supplement: Supplementary file 3 — Figure S1 [file 41419_2020_2438_MOESM3_ESM.tif]

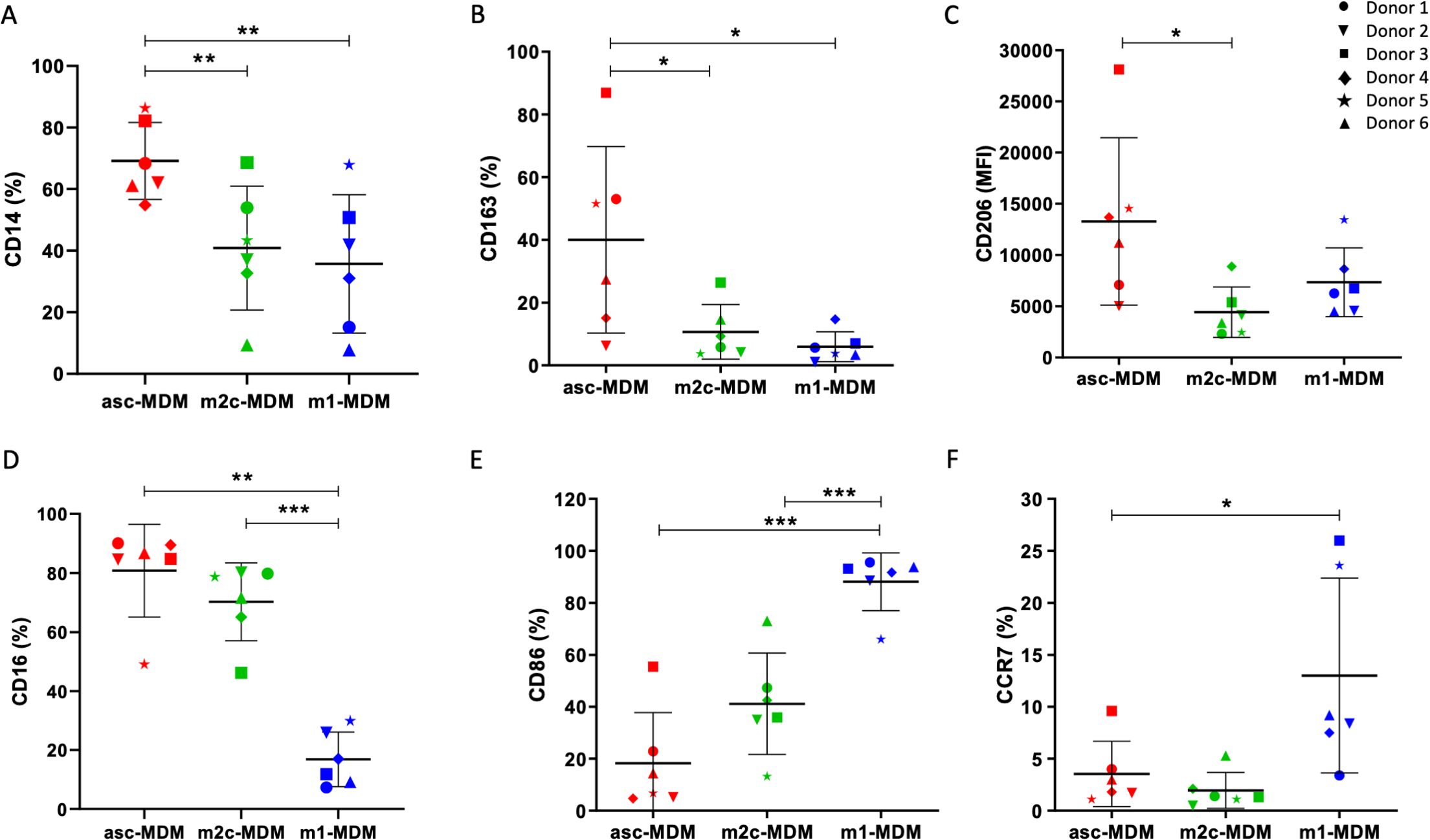

Supplement: Supplementary file 4 — Figure S2 [file 41419_2020_2438_MOESM4_ESM.tif]

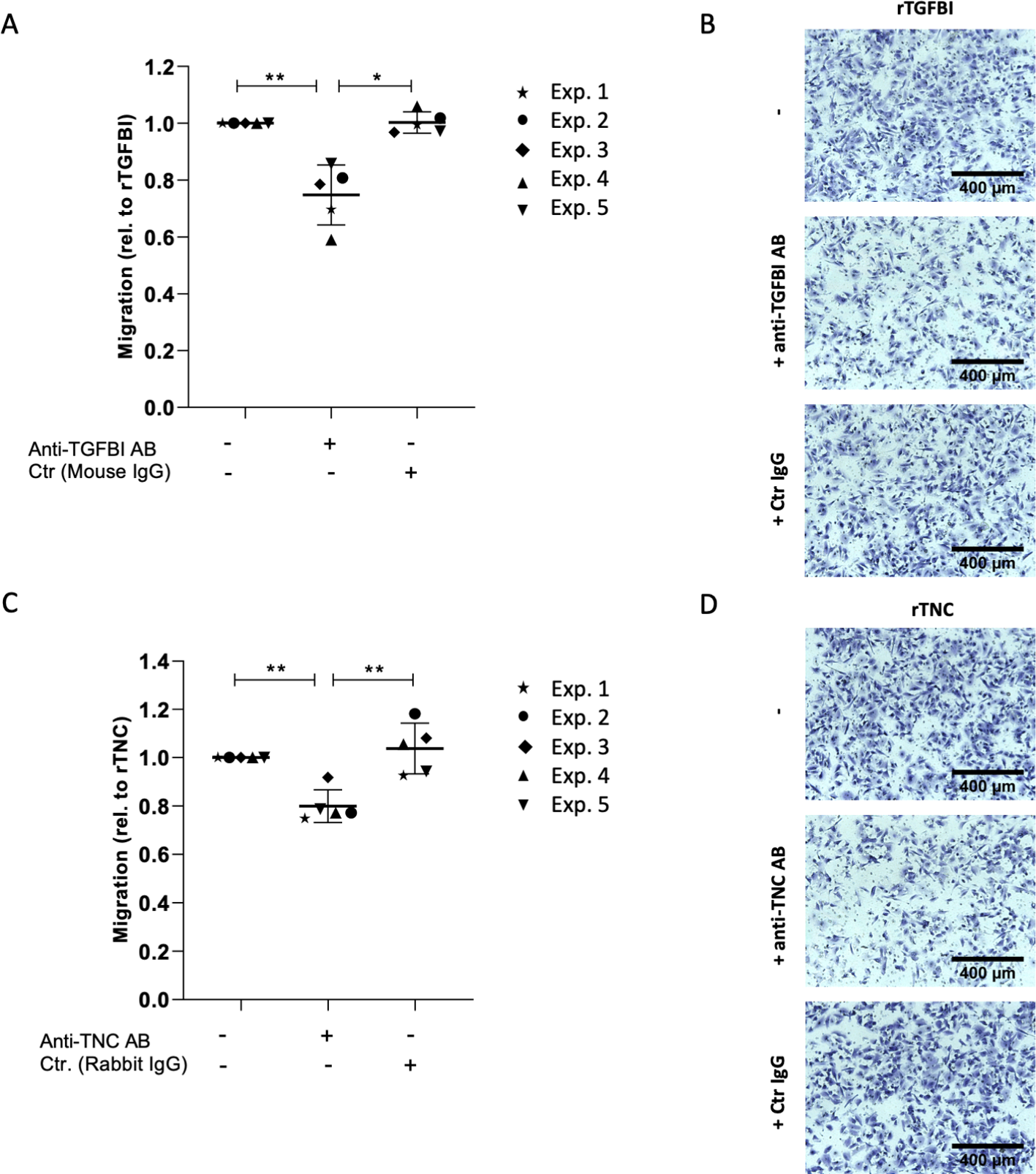

Supplement: Supplementary file 5 — Figure S3 [file 41419_2020_2438_MOESM5_ESM.tif]

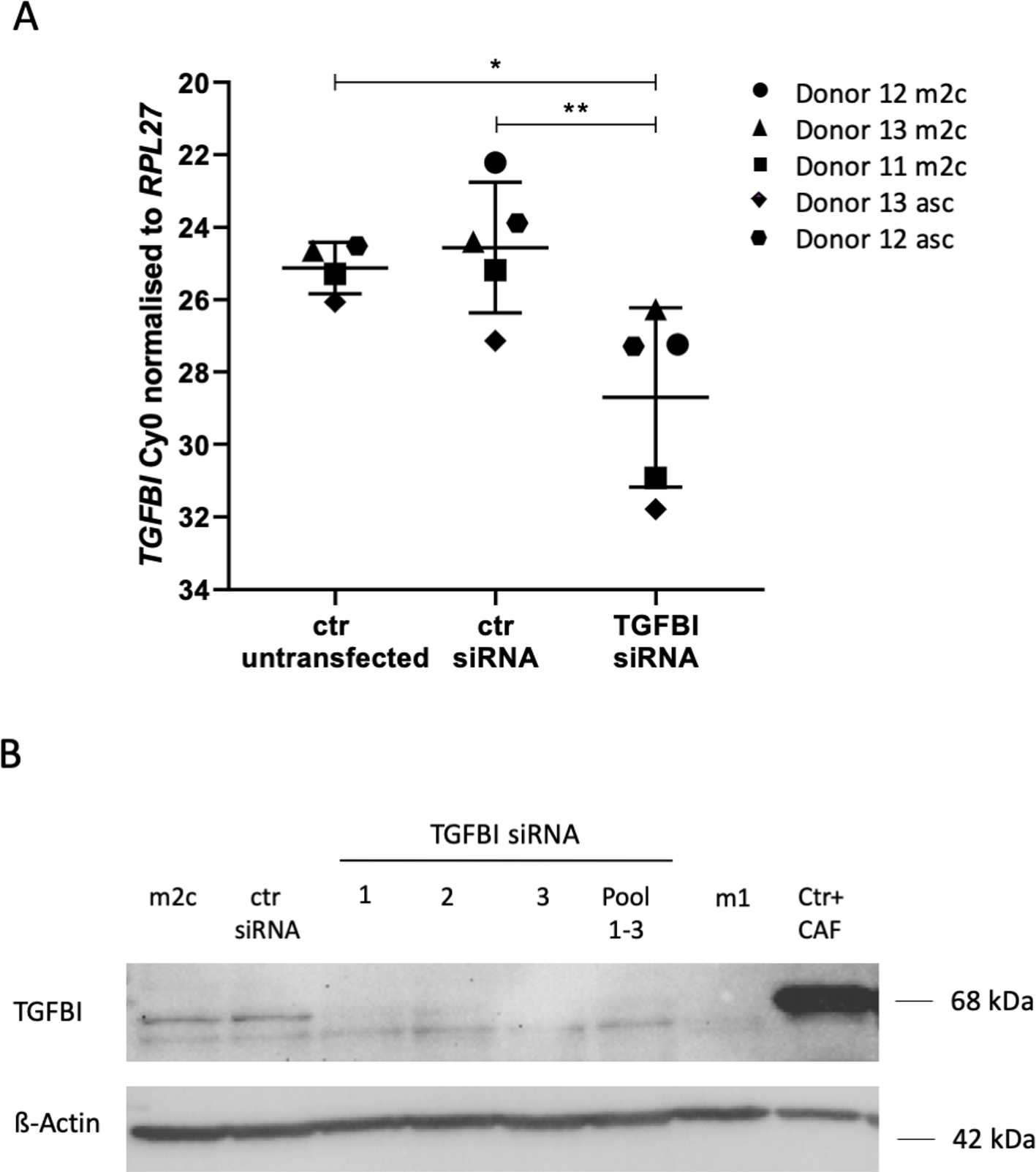

Supplement: Supplementary file 6 — Figure S4 [file 41419_2020_2438_MOESM6_ESM.tif]
