# Supplementary material for: Tumor-associated macrophages promote ovarian cancer cell migration by secreting transforming growth factor beta induced (TGFBI) and tenascin C
Source: Cell Death Dis. 2020 Apr 20;11(4):249. doi: 10.1038/s41419-020-2438-8 (PMC7171168; doi:10.1038/s41419-020-2438-8)
Supplement: Supplementary file 7 — Supplementary Figure Legends S1-S4 [file 41419_2020_2438_MOESM7_ESM.docx]

**Supplementary Figure Legends**

**Figure S1. Representative microscopic pictures of tumor cell migration induced by MDM secretomes as chemoattractant.** Conditioned media of m1-MDM, m2c-MDM and asc-MDM (donor 3) were applied as attractants for migration of OCMI tumor cell line OC_58 as described in Fig. 1D. Background migration in the absence of any attractant (Ctr-) as well as for FCS-induced migration (Ctr+) are included.

**Figure S2. Phenotypic characterization of MDM differentiation.** M1-MDM, m2c-MDM and asc- MDM from 6 donors were stained with antibodies to surface markers CD14 (A), CD163 (B), CD206 (C), CD16 (D), CD86 (E) and CCR7 (F) before used to study their impart on tumor migration (see Fig. 1). Expression was analyzed by flow cytometry to determine the percentage of positive cells and the mean fluorescence intensity (MFI). Depicted are expression data of donor-matched m1-MDM, m2c-MDM and asc- MDM. Horizontal lines indicate mean values. P-values were determined by two-sided, paired t-test (*p<0.05, **p<0.01, ***p<0.001).

**Figure S3.** **Inhibition of tumor migration-promoting activity of recombinant TGFBI and TNC by neutralizing antibodies.** Neutralization of tumor migration induced by rTGFBI (A, B) and rTNC (C, D). rTGFBI (0.5 µg/ml) and rTNC (EGFL repeat; 1µg/ml) were pre-incubated with 10µg/ml neutralizing anti-TGFBI and anti-TNC antibodies or species-matched IgG as controls for 1h before adding to OCMI cells (OC_58) for 17h. As a control, the cells were either left untreated or treated with the recombinant proteins without adding the antibodies. The OCMI cells were then allowed to migrate for 2h using FCS as chemoattractant in a transwell format. Migration is expressed relative to the migration induced by rTGFBI or rTNC alone. Depicted are the data of 5 experiments. Horizontal bars show the mean. P-values were determined by two-sided, paired t-test (*p<0.05, **p<0.01, ***p<0.001). Representative microscopic pictures of migrated cells induced by recombinant proteins in the presence and absence of neutralizing anti-TGFBI antibody (B) and anti-TNC antibody (D) are shown.

**Figure S4. Detection of TGFBI siRNA-mediated knockdown in macrophages on RNA and protein level.** (A) *TGFBI* transcripts were analyzed in m2c-MDMs and asc-MDM 48h after transfection with either control siRNA or TGFBI siRNA (pool of 3 siRNAs) by RT-qPCR with Cy0 values normalised to *L27*. The graph shows the results of 5 different experiments. Horiziontal lines indicate means. Two-sided, paired t-test was calculated for each TGFBI siRNA transfection (**p<0.01, ***p<0.001). (B) Detection of TGFBI expression in cell lysates of m2c-MDM transfected control siRNA or TGFBI siRNA (3 individual and pooled siRNAs) by Western Blot using anti-TGFBI antibody. ß-Actin was used as loading control. Untransfected m2c-MDM and CAFs known to express high levels of TGFBI were used as controls. Cell lysate from m1-MDM not secreting TGFBI was used as negative control. One representative Blot is shown for one knockdown experiment in m2c-MDM.
